# Current News

**Published:** 1897-07

**Authors:** 


### Current News.

### AMERICAN DENTAL ASSOCIATION.

THE Thirty-seventh Annual Session of the American Dental Association will be held at Old Point Comfort, Va., commencing at ten A.M., Tuesday, August 3, 1897.

GEORGE H. CUSHING,
*Recording Secretary.*

### NATIONAL ASSOCIATION OF DENTAL FACULTIES.

THE National Association of Dental Faculties will meet at Hygeia Hotel, Old Point Comfort, Va., Friday, July 30, 1897, at ten A.M. The Executive Committee will meet at nine A.M., Thursday, July 29; persons having business with this committee will please present their papers at the first session.

By order of

JONATHAN TAFT,
*Chairman Executive Committee.*

B. HOLLY SMITH,
*Secretary.*

---

### NATIONAL ASSOCIATION OF DENTAL EXAMINERS.

THE Fourteenth Annual Session will be held at Old Point Comfort, Va., commencing Friday, July 30, at ten A.M., and continuing in session Saturday, July 31, and Monday, August 2. The sessions will be held in the Hotel Chamberlin.

The hotels Hygeia and Chamberlin will give rates of $2.50 per day, two in a room; $3.50 per day, one in a room.

Fares on all the trunk lines one full fare and one-third, good for the sessions of the American and Southern Dental Associations.

The Old Dominion S. S. Co., Pier 26 North River, will sell excursion tickets, including meals and berths, $11.20, sailing every day at three P.M., from Thursday, July 29, using the name of the Secretary.

Let every State send its delegates!

CHARLES A. MEEKER, D.D.S.,
*Secretary.*

29 FULTON STREET, NEWARK, N. J.

---

### NEW JERSEY STATE DENTAL SOCIETY.

THE Twenty-seventh Annual Meeting will be held in the Grand Atlantic Hotel, Atlantic City, commencing on Wednesday morning, July 21, and continue in session two days. Seventeen papers by eminent men will be read upon interesting topics pertaining to the profession. Clinics of every description have been provided; four large rooms on the ground floor available for exhibits, with one-hundred-and-ten-volt current for electrical exhibits.

Hotel rates $2.50 per day up. Accommodations for seven hundred guests.

Friends from the West and East, contemplating attending the

American Dental Association, will be able to attend this meeting and take the Old Dominion Line of steamers from New York, Thursday, July 29, three P.M., at an excursion rate of $11.20, of the Pennsylvania Railroad rate of one and one-third fare.

CHARLES A. MEEKER, D.D.S.,
*Secretary.*

NEWARK, N. J.

---

### NEW ENGLAND ASSOCIATION OF DENTAL EXAMINERS.

ON the evening of March 4 the members of the various examining boards of New England dined at the University Club in Boston by invitation of the Massachusetts board. At this dinner, and at a conference after it, sixteen were present, representing Maine, Vermont, Massachusetts, Rhode Island, and Connecticut. It was the unanimous vote of those present that a permanent organization be effected to promote the following objects: To secure, through the co-operation of the various examining boards of New England, a high and uniform standard of qualifications for dental practitioners, and, so far as possible, uniformity of legislation, as well as uniformity in the requirements of candidates. A committee, consisting of Dr. John F. Dowsley, Dr. George L. Parmele, Dr. D. F. Keefe, Dr. T. J. Barrett, was appointed to frame a constitution and to call a meeting for organization.

This meeting was called together at Boston, June 3, and representatives from all the New England States were present except New Hampshire. The constitution presented by the committee was, after some minor changes, adopted, and the following officers were elected: President, John F. Dowsley, D.D.S., of Boston; Vice-President, D. F. Keefe, D.D.S., of Providence, R. I.; Recorder, George L. Parmele, M.D., D.M.D., of Hartford, Conn. The time and place of next meeting was left to the officers.

